# A qualitative evaluation of a co‐design process involving young people at risk of suicide

**DOI:** 10.1111/hex.13986

**Published:** 2024-02-11

**Authors:** Michelle Kehoe, Rick Whitehead, Kathleen de Boer, Denny Meyer, Liza Hopkins, Maja Nedeljkovic

**Affiliations:** ^1^ Department of Occupational Therapy Monash University Clayton Australia; ^2^ Alfred Health Melbourne Australia; ^3^ Alfred Mental and Addiction Health Melbourne Australia; ^4^ Centre for Mental Health and Brain Science Swinburne University Hawthorn Australia

**Keywords:** co‐design, process evaluation, youth suicide

## Abstract

**Background:**

Co‐design is becoming common practice in the development of mental health services, however, little is known about the experience of such practices, particularly when young people are involved.

**Objective:**

The aim of this study was to conduct a process evaluation of the co‐design which was undertaken for the development of an intervention for youth and adolescents at risk of suicide. This paper briefly outlines the co‐design process undertaken during a COVID‐19 lockdown and then focuses on a qualitative evaluation of the experience of taking part in a co‐design process.

**Setting and Participants:**

The evaluation involved young consumers of a public youth mental health service, their carers/parents and service delivery staff who had taken part in the co‐design process.

**Method:**

This study used follow‐up semistructured interviews with the co‐design participants to explore their experience of the co‐design process. Inductive thematic analysis was used to draw out common themes from the qualitative data.

**Results:**

It was found that despite the practical efforts of the project team to minimise known issues in co‐design, challenges centred around perceptions regarding power imbalance, the need for extensive consultation and time constraints still arose.

**Discussion:**

Despite these challenges, the study found that the co‐design provided a human‐centred, accessible and rewarding process for young people, parents and staff members, leaving them with the feeling that they had made a worthwhile contribution to the design of the new service, as well as contributing to changing practice in service design.

**Conclusion:**

With sensitivity and adaptation to usual practice, it is possible to include young people with suicidal ideation, their parents/carers and professional staff in a safe and effective co‐design process.

**Patient and Public Contribution:**

The authors would like to thank and acknowledge the young people with a lived experience and their carers who participated in the co‐design process and research evaluation component of this study. We also wish to thank the clinical staff, peer workers and family peer workers who participated in this research.

## 1 INTRODUCTION

Suicide remains the most common cause of death for people in Australia under the age of 25,^1^ with the number of young Australians dying from suicide doubling between 2009 and 2018.^2^ Suicide causes significant social, economic and epidemiological burdens, and developing effective methods for understanding, reducing and preventing suicide is a priority in Australia and elsewhere.^3^, ^4^ Government agencies and mental health services have recently sought to include the voices of consumers (those with a lived and living experience of mental health issues) and carers in the design, development and evaluation of suicide prevention initiatives.^4^, ^5^, ^6^ Their insights and expertise can assist in more effectively designing services that benefit consumers,^7^ however there are many potential challenges for services seeking to include young people at risk of suicide and their family members in a collaborative co‐design process.

### 1.1 Co‐design and co‐creation processes

Co‐design is used to engage a range of key stakeholders in a collaborative process where the expertise of multiple perspectives is acknowledged.^8^ More recently, the term co‐creation has been favoured to describe this process, however, in this paper the term co‐design has been used throughout.^5^ One of the main strengths of engaging in a co‐design process is that it addresses the problem of epistemic injustice in health services,^5^ in which the voice or ‘knowing’ of the consumer is often silenced or devalued.^9^ By involving the expertise of both mental health professionals and consumers, co‐design can go some way to addressing the traditional power imbalances experienced by consumers by incorporating and valuing multiple forms of expertise.^8^ By elevating the knowledge of both professionals and consumers equally, such processes can assist in collaboratively identifying problems and work towards co‐creating effective ways to address these problems.^10^

Although there are well‐documented benefits of co‐design and co‐creation, the experience of running co‐design programmes for service creation and implementation is not widely reported.^5^ There is, however, evidence to suggest that these programmes do not always run smoothly.^9^, ^11^, ^12^ In addition, the process of involving young people with mental health challenges in research and co‐design processes raises particular issues regarding burden, safety, power and competing interests.^11^

### 1.2 Co‐design challenges and benefits

Beyond the ethical and safety concerns, gaining personal views can be challenging, particularly when trying to engage hard‐to‐reach groups such as young people.^13^, ^14^ It is often the case that prior negative experiences with mental health services, such as loss of autonomy or exclusion from decision making about their own care, can result in young people being unwilling to participate in co‐design processes.^4^, ^10^, ^13^ Additionally, a power imbalance between researchers, clinicians, and consumers can impact the co‐design process,^15^ and this can result in an added layer of complexity when youth are involved.^14^, ^16^ Despite these challenges, the perspectives and views of individuals who have used or will use mental health services are increasingly valued in the development of new programmes.^13^

The facilitation of co‐design processes and the need to be wholly open to new ideas, perspectives and methods can be challenging for those already established in their models of operation and understanding of how things *should* operate.^17^ This can be a particular challenge when different parties use different communication strategies and key terms, resulting in barriers to effective collaboration and understanding.^8^, ^10^, ^12^

When the co‐design process works well, however, there are major benefits which include the creation of a more responsive service, increased involvement of consumers and carers, and a more democratic and inclusive approach.^18^ One area of mental health where co‐design tends to be particularly underutilised is suicide prevention, especially in the case of youth suicide.^4^ This is in no small part due to the sensitive and challenging nature of discussions involving suicide, suicidal ideation and suicide prevention, particularly with young people and their families.

### 1.3 Co‐design and suicide interventions

To address the underutilisation of consumer expertise, there has been a global push to include consumer input in the design, implementation and evaluation of suicide interventions,^4^ while taking into account the particular vulnerabilities of this cohort, with the aim of making suicide‐prevention services less stigmatising, more accessible, and more tailored to the specific needs of those requiring support,^17^ thereby encouraging help‐seeking and improving programme effectiveness. From the limited research into the use of co‐design for the development of suicide prevention programmes, it has been found that outcomes tend to be positive, especially in cases where the roles of external stakeholders are more collaborative rather than merely consultative or advisory.^4^ For example, Pocobello et al.^19^ found that when mental health services were truly co‐designed and co‐produced, service users had less reliance on medication and fewer hospitalisations than individuals engaging in traditional mental health services.

Despite there being some research on the use of co‐design with adults in suicide intervention programmes, there has been considerably less consultation with young people in the design of these interventions.^5^, ^14^, ^20^ A recent meta‐analysis^20^ into suicide prevention among young people showed that out of 99 studies analysed, none included young consumers in the design or creation of interventions. However, one study that did engage young people in the co‐design of suicide prevention was conducted by Thorn et al.^14^ The researchers in that study co‐created safety protocols with the help of young people for social media‐based discussions about suicide. Their research highlighted the specific sensitivities and challenges that need to be navigated when working with young people when co‐designing services to address suicide. In particular, the need to develop specific safety protocols to ensure no harm was caused while dealing with potentially challenging content was emphasised. The protocols included tailored wellness plans to support the young people, access to a psychologist for the young people, and, following participation, young people were contacted to check on their mental health and gauge any need for support. Results of the study found that the majority of young people involved in the co‐design process felt it was enjoyable and beneficial, and that participating made them feel more confident in supporting other people at risk of suicide.^19^

### 1.4 Background information on the co‐design process evaluated in this study

The current study was based on a co‐design process carried out to develop a new, postsuicide support programme for young people. It was conducted at the height of the COVID‐19 pandemic, in Melbourne, Victoria, Australia. The programme was one of four newly funded pilot programmes in different child and youth mental health services and followed the successful implementation of a similar service for adults.^21^ The co‐design process sought to include features of the adult model, such as providing intense, psychosocial outreach support, while seeking new perspectives regarding the adaptations required to meet the specific needs of young people. Hence, the main aim of the co‐design process was to design a new programme, within an existing tertiary child and youth mental health service, where children and young people could be referred after a suicide attempt, self‐harm or with persistent suicidal ideation. This process was conducted in alignment with broad co‐design philosophy to ensure the expertise of lived experience was valued alongside other perspectives, and to limit epistemic injustice often prevalent among health services.^6^

#### 1.4.1 Co‐design participants

Those invited to participate in the co‐design were either young people who were prior users of the mental health service or the parent or carer of a young service user, as well as staff employed at the mental health service and local community partners who had contact with the mental health service.

The co‐design process was supported by an external group facilitation expert and a research team. Within the mental health service, the project was supported by a co‐design project team that consisted of a project officer, youth peer worker, family peer worker and clinical staff member. Young people and family members could participate either via a one‐on‐one interview or attendance at one or both online group workshops.

#### 1.4.2 Co‐design procedure

The two workshops, each of 2.5 h duration, were conducted during August and September 2021 via Microsoft Teams (MS Teams) using Miroboard^©^, a whiteboard‐style online tool for capturing participant responses (see www.miro.com). Those who participated could either add a virtual ‘sticky note’ (similar to a ‘post‐it note’) to capture their thoughts or, if they were unable to navigate the technology, there was a research team member/scribe available to capture their feedback.

The focus of workshop 1 was to obtain participants' perspectives of what components they felt would be important for the proposed postsuicide support service. At the end of this workshop, the data were collated and populated on the Miroboard^©^ to develop a programme proposal. Workshop 2 was conducted with the objective of getting feedback about the proposed programme. Following workshop 2, the plan for the new programme was able to be refined based on the participants' feedback.

#### 1.4.3 Co‐design practical and ethical considerations

The time of day for the workshops was chosen for the convenience of those attending. For the young people late morning was recommended to allow for any ill‐effects from medication to wear off. For carers/parents, there was a need for a similar time so as not to impact school‐age children in the family. The dates for the workshops needed to comply with the funding body's timeline, hence the need to hold them during the height of the pandemic‐induced lockdowns.

*Safety and power*: Consideration was given to the safety of all participants during the co‐design process due to the sensitive nature of the topic and the vulnerability of the young consumers. As an alternative to the workshops as described, participants were offered the option to provide their feedback in writing, in a one‐on‐one conversation, or via direct messages to a co‐design project team member during their participation in the workshops. During the workshops, a psychologist was on standby via phone for all participants, but in particular for young consumers, in the event they found the content distressing. This offered a sense of reassurance for the co‐design project team as well as the young consumers, however, this option was not utilised.

Before each workshop, the young consumers met individually on MS Teams with the youth peer worker who was a member of the co‐design team. At the end of each workshop, the young consumers and parent/carer participants were contacted by either the youth peer worker or the family peer worker to debrief. To reduce any power imbalance, the young consumers, carers/parents and clinician/community partners were placed into three different breakout rooms during the workshops, so that they could discuss their ideas separately before returning to the larger group.

The outcomes from the co‐design process were collated and documented. These outcomes are reported elsewhere.^21^

### 1.5 Aim and research questions for this study

The aim of this study was to conduct a qualitative process evaluation of the above co‐design process, using the Medical Research Council guidelines for good research practice, with an evaluation framework focussed on the fidelity of the process, the main mechanisms driving the outcomes as well as the contextual factors impacting the process.^22^ In particular, the evaluation sought to address the following questions which were of specific interest in regard to the process by which the co‐design was undertaken:

1. To what extent was the co‐design process successful in eliciting the views of young people, families, stakeholders and staff in informing the design and development of a youth suicide intervention programme.
2. In what ways do co‐design processes need to be adapted to meet the needs of vulnerable young people to ensure their safety?

Because the evaluation was very specific to the context of the co‐design, open‐ended interview questions were considered to be the best way of eliciting participants' views. The methods, results and implications for this process evaluation are provided below.

## 2 METHODS

This section describes the methods used to evaluate the above co‐design process. This evaluation started 6‐months after the co‐design workshops and individual interviews were completed. The data collected from one‐on‐one interviews is used to identify key themes that illustrate participants’ experience of participating in this co‐design process.

### 2.1 Participants and procedure

All participants of the co‐design workshops were contacted via phone or email by a member of the research team, inviting them to participate in the evaluation of the co‐design process. One young consumer, one parent/carer and three staff members agreed to participate in the evaluation. A semistructured interview guide was developed based on research into co‐design and what constitutes a successful co‐design process.^14^, ^19^ An example of the broad interview questions is shown in Appendix A. Questions were designed to allow the participants (young person, parent or staff) to share their own personal views on the process so that inductive thematic analysis could be undertaken without bias or prejudgement. Within the broad questions, further probes around known issues in co‐design were included, such as power imbalance, inclusivity and feeling heard throughout the process. In addition, the guide sought to elicit views of how the co‐design process was managed and to what extent the needs of the various stakeholders were satisfied. This was done to capture perceptions of what constituted a successful co‐design process and any perceived barriers or challenges (see Appendix A for the interview guide). The evaluation interviews were conducted by telephone or videoconferencing with a duration of 15–60 min and an average time of 40 min.

### 2.2 Data analysis

To identify key themes within the data, a thematic analysis was conducted on transcripts of the interviews. Thematic analysis was ascertained to be an appropriate form of analysis to inductively identify themes grounded in participant data.^23^, ^24^ Specifically, the analysis followed Braun and Clarke's^23^, ^24^ recommendations for a six‐stage thematic analysis, with coding reliability approaches utilised to limit any individual bias from those analysing the data.^24^ Interview data were independently coded by the research team members. Initially, each member of the research team became familiar with all responses before generating codes which were refined to create themes. The coding underwent checking by another member of the research team (MK, RW, KD, DM, LH, MN) and discrepancies were discussed until agreement was reached. Subsequently, two researchers (MK, RW, KD, DM, LH, MN) collated the themes and extracted key quotes to illustrate the themes. See Table 1 for the analysis process.

**Table 1:** The analysis process.

Analysis phase	Description of the phase	Data example
1. Becoming familiarised with the data.	This phase involved conducting the one‐to‐one interviews with participants in the co‐design and the interviewers, noting any points of interest. The transcripts of these interviews were then read by the research team with any potential themes noted.	
2. Generating preliminary codes	The transcripts of the interviews were reread and data that was deemed to capture individuals’ experiences of taking part in the co‐design was initially colour coded into preliminary themes. Themes were entered into a thematic table.	Initial quote: ‘And I feel like it was very dominated by either the carers or the clinicians, not the lived‐experience person, if that makes sense… And it's kind of hard on Zoom as well’ (quote from young person)’ initial code: Challenge of being on zoom
3. Searching for themes	The preliminary coding was then discussed between members of the research team and themes were identified that loosely aligned with the questions asked in the interview (e.g., power imbalances, feeling supported).	Relevant interview question for above quote: Can you describe the extent to which you felt valued and supported? Prompt. Did you feel listened to and heard? If not valued why not? Refined coding for above quote: Power and safety and online challenges
4. Reviewing themes	Themes identified in phase three were reviewed and cross‐referenced in relation to data from the thematic tables.	Supporting secondary quote for above coding: ‘I think sometimes I felt some of the professionals were talking too much and not listening enough… I could see some people were about to talk and they never got a chance to say anything’ (quote from parent)
5. Defining and naming themes	Further refinement of themes was conducted, and the meaning and definitions were discussed among the research team.	Refined theme for above coding: Addressing power
		Example subthemes
		1. allowing opportunity to share, 2. filtering and 3. guidance around the process.
6. Producing a thematic report	This involved reading through the thematic table and selecting the extracts that best capture the themes present. This phase also involved a final discussion of themes and extracts used, with some extracts removed from the report.	Example quote (filtering): ‘[there was] filtering of what people said by the scribes (via post‐it notes)’ (member of co‐design team)

## 3 RESULTS

**Figure 1:**
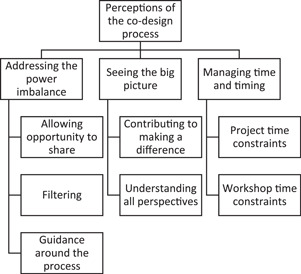
Superordinate main themes with subordinate themes.

The analysis of these interviews indicated that three main themes regarding participant perceptions of the co‐design process could be identified together with underlying subthemes. The superordinate themes were: Addressing the power imbalance, seeing the big picture, and managing time and timing. Figure 1 illustrates the main themes and their subthemes.

### 3.1 Addressing the power imbalance

Although the project team had made a great deal of effort to ensure that any potential power imbalance was addressed, some of the participants noticed a subtle imbalance of power within the co‐design process. Within this power dynamic, participants articulated the ways in which this dynamic impacted either positively or negatively on their participation, as highlighted in three subordinate themes:

1. allowing opportunity to share,
2. filtering and
3. guidance around the process.

Quotes relating to these themes are presented in Table 2.

**Table 2:** Illustrative data for superordinate theme ‘addressing the power imbalance’.

Subordinate theme	Data
Allowing opportunity to share	*Quote* 1. I remembered one of the other things that was reflected by the young people was that having the group [discussion] with carers and consumers sometimes felt difficult for them [the young person] to speak freely [in the broader group]… I think had an imbalance of consumer/user as there were no shows on the day. That did change how we did round two of the workshops, so we gave more space for each group to speak freely within their own cohort. *Quote* 2. And I feel like it was very dominated by either the carers or the clinicians not the lived‐experience person, if that makes sense. (young person) *Quote* 3. Trying to genuinely respect and value the lived experience and bring that, really highlight, that lived experience, rather than going, ‘We've got these ideas. Is that okay with you?’. (member of co‐design team)
Filtering	*Quote* 4. [there was] filtering of what people said by the scribes (via post‐it notes). (member of co‐design team) *Quote* 5. I think there were some ideas that were not, again because of the facilitator, not explored enough. (parent) *Quote* 6. …that filtering process about what they may have told me on [previous] talks whether they felt … if they weren't able to participate in those online spaces they still have an opportunity to chat to me. (member of co‐design team)
Guidance around the process	*Quote* 7. It's a very good idea to have [name], a peer‐worker reaching out to us rather than a clinician or, I don't know … It just feels more genuine because they really did put thought into the conversations … doing this for us [other young people]. (young person) *Quote* 8. All of the professionals who were guiding it, I think they did a wonderful job. (parent)

The first subordinate theme relating to power imbalance concerned the lack of opportunity to share (see Table 2 Quote 1). One parent described the imbalance they saw as follows: ‘sometimes I felt some of the professionals were talking too much and not listening enough… I could see some people [referring to young consumers and parent/carers] were about to talk and they never got a chance to say anything’. One young person felt there was an imbalance due to dominant carers or clinical staff (Table 2, Quote 2). However, this was in contrast to members of the co‐design team, who felt they genuinely tried to address the power imbalance (Table 2, Quote 3).

The second subordinate theme, which also tapped into the power dynamic, was an issue around ‘filtering’. Filtering comprised either how participants felt their view was ‘filtered’ by others or how they chose to filter their own response (Table 2, Quote 4). The parent participant also felt there was filtering by the facilitator who did ‘not explore’ ideas fully. However, in contrast, one member of the co‐design team felt that filtering was occurring due to the level of comfort a participant may or may not have felt in sharing their views (Table 2, Quote 6). This theme highlights the intended desire to successfully engage stakeholder views, as well as the difficulty in always being able to do so.

The third subordinate theme related to the guidance and support provided by the co‐design project team. Despite the issues described around power, the participants felt the space was safe due to the way the co‐design project team was constituted. In particular, the young person described their experience of having authentic conversations with a youth peer who was part of the co‐design project team (Table 2, Quote 7): The young person went on to say they felt the youth peer worker was genuinely ‘doing this for us [other young people]’. The parent participant also valued the guidance of the co‐design team (Table 2, Quote 8).

### 3.2 Seeing the big picture

The second superordinate theme concerned a ‘big picture’ participant view of the co‐design process with two subordinate themes:

1. contributing to making a difference and
2. understanding all perspectives.

Quotes relating to these themes are presented in Table 3.

**Table 3:** Illustrative data for superordinate theme ‘seeing the big picture’.

Subordinate theme	Data
Contributing to making a difference	*Quote* 1. It's rewarding to be able to talk about these things and knowing that you might be contributing to something bigger. (young person) *Quote* 2. It's so nice to be heard… and it's just nice that someone is trying to make a difference with all of this. (parent)
Understanding all perspectives	*Quote* 3. Everybody was really stepping into seeing everybody's perspective all at once. (member of co‐design team)

Participants indicated that their reason for contributing to the co‐design workshops was so they could make a difference in the lives of others. This participation provided its own reward (Table 3, Quote 1). In addition, their participation in the co‐design process gave them a better understanding of what the ‘big picture’ entailed allowing them to hear and value different views as well as feeling heard in expressing their own views (Table 3, Quote 2).

The second subordinate theme under ‘the big picture’ related to the holistic understanding acquired, with everyone making an effort to see and understand the views of others (Table 3, Quote 3). Participation in the workshop was seen as ‘very rewarding and (a) great experience, and you get to talk to other people and get so many different perspective’ (parent).

### 3.3 Managing time and timing

The final theme was around timing and the amount of time needed to meet the co‐design requirements of the funding body.

Quotes relating to this theme are presented in Table 4, under two subordinate themes:

1. project time constraints and
2. workshop time constraints.

**Table 4:** Illustrative data for superordinate theme ‘managing time and timing’.

Subordinate theme	Data
Project time constraints	*Quote* 1. [There were] an awful lot of meetings where we're teaching the same things over and over again, whether it was with the department, whether it was the design team, or even internally. Um, and that felt, at times, that, that was really slow progress. (member of co‐design team) *Quote* 2. Realising you can't [make decisions] because it's got to go to this consultation and that consultation and they're [the funding body] not going to agree, and they have a different opinion. (member of co‐design team) *Quote* 3. There were times when we really wanted to do things, but we were told we couldn't, or we shouldn't. (member of co‐design team)
Workshop time constraints	*Quote* 4. Everybody had a few things that they would like to have said (but did not get the opportunity). (parent)

The issue of managing time and timing constraints was experienced in contrasting ways by the co‐design project team and other co‐design participants. For the co‐design project team members, this was experienced as the extent and degree of consultation required resulting in repetition, multiple meetings, and slow progress (Table 4, Quote 1). There were also limitations placed on the co‐design project team which were beyond their control. This created a tension between the ideas that were shared in the co‐design and the constraints of the funding body around what was achievable (Table 4, Quotes 2 and 3). As a result, the co‐design team felt that ‘the process was frustrating’. The co‐design team members attributed some of the frustration and delays to the external facilitation expert who was not familiar with the service and was ‘very hands off’ and ‘they had no knowledge … [and] would segue [transition] into things which were never going to be achievable’. Such delays had the potential to cause a ripple effect and impact on the efficiency of the co‐design process.

Time was also an issue for workshop participants although with a different focus. A parent participant felt there was ‘not enough time’ and they would have appreciated more opportunities to share their thoughts (Table 4, Quote 4). Thus, it is clear that managing a process that, by its very nature required extended consultation in a restricted timeframe, resulted in a degree of frustration for both members of the co‐design team and workshop participants.

## 4 DISCUSSION

This study undertook an examination of the experience of taking part in a participatory co‐design process aiming to design a new programme to support suicidal young people. The findings of the study highlight the power imbalance and frustrations encountered during such a co‐design process, as well as the benefits of this process and the efforts needed to ensure the process works successfully. Young people and parents described the benefit of taking part as feeling valued and heard, and helping them to believe that they could ‘make a difference’. Staff member participants described the benefits of seeing a bigger picture and understanding different perspectives. There are some key implications from the findings of this study.

### 4.1 Involving young people in co‐design

Although there is support for a co‐design process in service development it is not without its critics, in particular in relation to the wellbeing of the lived experience participants.^25^ This is a particular challenge when involving young people due to ethical concerns, reports of negative youth experiences, and heightened risk of power imbalance.^13^ Despite these challenges, this study found that young people valued the experience and opportunity, which gave them a sense of contributing to the lives of others in the future. The efforts of the co‐design project team to ensure the safety of participants were appreciated by the young people, and the effectiveness of these measures can be seen in the way back up safety plans, such as having a psychologist on‐call during the workshops, were not needed.

### 4.2 Creating safety

Due to potential ethical concerns when engaging young people in co‐design, especially those with a lived experience of suicidality, a safe, supportive and engaging environment is critical.^14^ For the current study, this was of particular importance at a time when online workshops were the only way to conduct workshops, due to the COVID‐19 pandemic lockdowns. This was achieved through the provision of individual support before and after the workshops, the use of breakout rooms for ensuring communication within ‘like’ groups (e.g., young people separate from parents/carers), and on‐hand professional staff, for anyone feeling distressed during the workshops, (see Section 1.4.3). In addition, there was the option of a one‐on‐one interview rather than workshop attendance for anyone who was not comfortable with the online workshop format. This approach was considered as being supportive and genuine. Both young people and parents alike appreciated the thoughtfulness and sensitivity with which the co‐design project staff ensured that their needs were met. Although there are often additional financial costs incurred in creating individualised processes and protocols to ensure safety, these are necessary when undertaking research around the topic of suicide, particularly when working with young people.^14^ The reports from parents and young consumers indicated that the sensitivity and genuine nature of the team made this a safe and rewarding process. This highlights the need to tailor co‐design activities specific to each project and the participants involved.^26^

### 4.3 Reducing power imbalance

Despite a concerted effort and creative approaches by the co‐design team to address any power imbalance during the co‐design workshops, there was still a range of perceptions as to how successfully this was achieved. Part of this can perhaps be attributed to the online nature of the workshops. In particular, it was difficult to ensure that all participants got to participate fully in this environment and the voice of lived experience was sometimes incorrectly filtered by facilitators and scribes. Interestingly, the power imbalance described by some participants was not always recognised by the co‐design project team. This highlights the importance of understanding different perspectives of power and being aware that power dynamics may be implicit, thereby requiring ongoing awareness of potential imbalances throughout the co‐design process. Nevertheless, participants expressed an appreciation for the professional guidance provided by the facilitators and for the opportunity to share.

### 4.4 Conflicting expectations

There were some challenges highlighted through this project that are likely to occur in any co‐design process, especially when decisions are imposed from outside of the organisation hosting the co‐design process. These challenges related to timelines, the time allocated, staffing, and procedures. The varying expectations from a range of stakeholders with a diverse understanding of the co‐design process, the model of operations and its implementation may contribute to further challenges. This situation can be exacerbated when communication and key terms around aspects of a co‐design project are unclear, resulting in barriers to successful understanding.^8^, ^12^

For co‐design to be undertaken in an effective manner the process needs to be thorough, with pre‐workshop scoping and adequate allocation of time. Effective co‐design requires a process that ensures that all those who participate are clear on the parameters, so their contribution is felt to be of value^4^ and not, as with many co‐design processes, ‘tokenistic’ or ‘consultative’ as opposed to ‘true’ co‐design.^4^, ^5^

Due to the growing importance of co‐design in the field of service development and re‐development, and the fact that funding is often tied to a co‐design process, it is important for the process to be more than just a low‐impact, superimposed, novelty activity to fulfil expectations and tick boxes.^12^ This study highlights the need to ensure that there is a true willingness on behalf of the implementing service to challenge preconceived ideas, be open to new knowledge and processes, and actively seek collaboration. If a process truly embodies the ethics and philosophy of co‐design as a power‐leveller, promoting shared knowledge and epistemic justice, and facilitating the voices of consumers and community members, true co‐creation should be achievable, resulting in the development of a more suitable and effective service for consumers.^11^

## 5 CONCLUSION

Government commitment and funding have gone some way to addressing the increase in the need for youth mental health services, however, it is important to ensure that any mental health interventions and programmes meet the requirements of those who are users of these services. The co‐design process evaluated in this study provided important information regarding the inclusion of vulnerable young people in the design of such services.

Participation in this co‐design process was feasible, safe and valued by those who participated. Evaluation of the process has highlighted the need to adapt the co‐design process when working with suicidal young people, with greater attention paid to safety, appropriate communication and professional guidance. When the co‐design process involves online workshops, these are particularly important challenges. In addition, the study highlights that it is not possible to use a ‘one‐size’ fits all approach to co‐design. The competing expectations of stakeholders mean that difficult compromises will often be necessary.

The need for co‐design and co‐creation will continue with the growing recognition that service users should and need to be involved in the design and development of the services that they use.^11^ To be considered as ‘genuine’, a co‐design process needs to be tailored to the specifics of a project and it needs to address the unique and sometimes complex needs of individual cohorts. This evaluation study has highlighted a range of needs that must be addressed by the co‐design team to create a supportive environment while balancing the conflicting demands placed upon them. There is often a sensitivity in the inclusion of the consumer voice, especially that of young people. However, this study shows that including the views of young people in the design of a suicide prevention service can be achieved successfully. This can also be a beneficial experience for the young people involved, allowing them to better understand the service requirements and to feel that they are making a valuable contribution to the broader system and community.

## AUTHOR CONTRIBUTIONS

**Michelle Kehoe**: Conceptualisation; investigation; funding acquisition; writing—original draft; methodology; writing—review and editing; project administration; formal analysis; data curation; validation. **Rick Whitehead**: Writing—review and editing; formal analysis; data curation; validation. **Kathleen de Boer**: Writing—review and editing; validation; formal analysis. **Denny Meyer**: Funding acquisition; writing—review and editing; investigation. **Liza Hopkins**: Validation; writing—review and editing; formal analysis; project administration. **Maja Nedeljkovic**: Writing—review and editing; validation; formal analysis.

## CONFLICT OF INTEREST STATEMENT

The authors declare no conflict of interest.

## ETHICS STATEMENT

Ethical approval for the study was obtained from the Alfred Health and Swinburne University Human Research Ethics Committees. All participants were older than 17 years of age and provided written consent. Note on language: In this paper, the terms ‘consumer’ and ‘young consumer’ are used to describe a person or young person with lived or living experience of mental health issues. The authors acknowledge that other terms are used such as a person with lived and living experience and service user in different countries and jurisdictions. Those who care for the young person are referred to as parents, families and carers but we acknowledge that other terms may be used for unpaid carers throughout the world.

## Data Availability

The data that support the findings of this study are available from the corresponding author upon reasonable request.

## APPENDIX A POST CO‐DESIGN INTERVIEW GUIDE

- How did you become involved in the co‐design workshop? For example, what role did you come to the co‐design workshop with?
- Can you explain how you found the experience of participating?
  ○ Prompt. What was the best part? What was the least enjoyable part (if anything)?
- Can you describe the extent you felt valued and supported being a part of the process? Prompt. Did you feel listened to and heard? If not valued why not?
- What challenges do you think there were about the co‐design process? What ways, if any were these challenges overcome?
- Do you think the ideas you discussed in the co‐design were listened to and taken on board? Elaborate on why or why not
- How did you find the technology? Prompt—what was easy/hard?
- If anything should or could be done differently in the future what would that be and why? Prompt. What lessons could be learnt?
- Is there anything else you would like to tell me about your participation in the co‐design workshop?

Thank you for your time
